# DDBJ update in 2023: the MetaboBank for metabolomics data and associated metadata

**DOI:** 10.1093/nar/gkad1046

**Published:** 2023-11-16

**Authors:** Takeshi Ara, Yuichi Kodama, Toshiaki Tokimatsu, Asami Fukuda, Takehide Kosuge, Jun Mashima, Yasuhiro Tanizawa, Tomoya Tanjo, Osamu Ogasawara, Takatomo Fujisawa, Yasukazu Nakamura, Masanori Arita

**Affiliations:** Bioinformation and DDBJ Center, National Institute of Genetics, Mishima, Shizuoka 411-8540, Japan; Bioinformation and DDBJ Center, National Institute of Genetics, Mishima, Shizuoka 411-8540, Japan; Bioinformation and DDBJ Center, National Institute of Genetics, Mishima, Shizuoka 411-8540, Japan; Bioinformation and DDBJ Center, National Institute of Genetics, Mishima, Shizuoka 411-8540, Japan; Bioinformation and DDBJ Center, National Institute of Genetics, Mishima, Shizuoka 411-8540, Japan; Bioinformation and DDBJ Center, National Institute of Genetics, Mishima, Shizuoka 411-8540, Japan; Bioinformation and DDBJ Center, National Institute of Genetics, Mishima, Shizuoka 411-8540, Japan; Bioinformation and DDBJ Center, National Institute of Genetics, Mishima, Shizuoka 411-8540, Japan; Bioinformation and DDBJ Center, National Institute of Genetics, Mishima, Shizuoka 411-8540, Japan; Bioinformation and DDBJ Center, National Institute of Genetics, Mishima, Shizuoka 411-8540, Japan; Bioinformation and DDBJ Center, National Institute of Genetics, Mishima, Shizuoka 411-8540, Japan; Bioinformation and DDBJ Center, National Institute of Genetics, Mishima, Shizuoka 411-8540, Japan

## Abstract

The Bioinformation and DNA Data Bank of Japan (DDBJ) Center (https://www.ddbj.nig.ac.jp) provides database archives that cover a wide range of fields in life sciences. As a founding member of the International Nucleotide Sequence Database Collaboration (INSDC), DDBJ accepts and distributes nucleotide sequence data as well as their study and sample information along with the National Center for Biotechnology Information in the United States and the European Bioinformatics Institute (EBI). Besides INSDC databases, the DDBJ Center provides databases for functional genomics (GEA: Genomic Expression Archive), metabolomics (MetaboBank) and human genetic and phenotypic data (JGA: Japanese Genotype-phenotype Archive). These database systems have been built on the National Institute of Genetics (NIG) supercomputer, which is also open for domestic life science researchers to analyze large-scale sequence data. This paper reports recent updates on the archival databases and the services of the DDBJ Center, highlighting the newly redesigned MetaboBank. MetaboBank uses BioProject and BioSample in its metadata description making it suitable for multi-omics large studies. Its collaboration with MetaboLights at EBI brings synergy in locating and reusing public data.

## Graphical Abstract

**Graphical Abstract:**
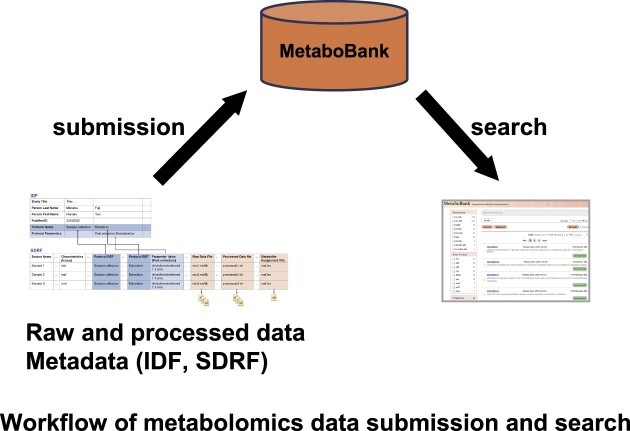

## Introduction

The DNA Data Bank of Japan (DDBJ) is a public database of nucleotide sequences at the Bioinformation and DDBJ Center (DDBJ Center; https://www.ddbj.nig.ac.jp) of the National Institute of Genetics (NIG) (1). Since 1987, the DDBJ has been accepting annotated nucleotide sequences, issuing accession numbers, and distributing them in collaboration with GenBank at the National Center for Biotechnology Information (NCBI) (2) and the European Nucleotide Archive (ENA) at the European Bioinformatics Institute (EBI) (3). This collaborative framework is known as the International Nucleotide Sequence Database Collaboration (INSDC) (4).

Within this INSDC framework, the DDBJ Center has been maintaining the DDBJ Sequence Read Archive (DRA) for raw sequencing data and alignment information generated from high-throughput sequencing platforms and analysis pipelines (5), the BioProject database for study information, and the BioSample database for sample information (1,6). This comprehensive biological data resource enriched with research contexts is guaranteed free and access-unrestricted by the INSDC standard (7). In addition to these resources, the DDBJ Center maintains the Genomic Expression Archive (GEA) (8) for quantitative data from functional genomics experiments (e.g. gene expression and epigenetics) as a counterpart to the Gene Expression Omnibus at NCBI (9) and the ArrayExpress at EBI (10).

For controlled-access information, the DDBJ Center provides the Japanese Genotype–phenotype Archive (JGA) to store and distribute human genotype and phenotype data resulting from biomedical research (11,12). JGA is operated in collaboration with the National Bioscience Database Center (NBDC, https://biosciencedbc.jp/en/) at the Japan Science and Technology Agency (JST), which reviews data submission and grants access under its guidelines for sharing human data (https://humandbs.biosciencedbc.jp/en/guidelines). JGA is a collaborating counterpart of the major controlled-access databases, the database of Genotypes and Phenotypes (dbGaP) at NCBI (13) and the European Genome–phenome Archive (EGA) at EBI (14).

A recent demand is the integration with other ‘omics’ technologies such as metabolomics, which systematically identifies and quantifies small compounds in biological systems (15). Being a study on phenotypic building blocks, metabolomics contributes to biomarkers and pharmaceutical research, nutrition and toxicology, systems biology and metabolic engineering (16). We launched the original MetaboBank in late 2020 as a public repository (1), but for easier analysis and integration, its data model and submission format were completely redesigned in September 2021. Now metadata are described in a structured and standardized MicroArray Gene Expression Tabular (MAGE-TAB) format (17) for compatibility with the functional genomics data in GEA and ArrayExpress. This format is used by the proteomics database PRIDE at EBI (18), and its derivative format ‘ISA-TAB’ is used by the metabolomics database MetaboLights at EBI (19,20). Another update was cross-referencing the BioProject and BioSample databases to link with genomics and transcriptomics data in INSDC.

To operate the above archival databases, the DDBJ Center maintains the NIG supercomputer and also lets researchers in Japan login and analyze our public data resources. The supercomputing system has recently enhanced its storage to accommodate the growing demand.

In this article, we report updates to the databases and services of the DDBJ Center, highlighting the new repository: the MetaboBank. All resources are available at https://www.ddbj.nig.ac.jp and the data are downloadable at ftp://ftp.ddbj.nig.ac.jp and https://ddbj.nig.ac.jp/public/.

## DDBJ archival databases

### Data contents: unrestricted- and controlled-access databases

In 2022, DDBJ accepted 6036 submissions for nucleotide sequences, among which 74.2% were contributions from domestic Japanese research groups. The DDBJ has released all public DDBJ/ENA/GenBank nucleotide sequence data periodically in a flat-file format. The latest release of June 2023 contains 3 639 350 806 sequences and 24 306 833 885 555 bp, and the DDBJ contributed 5.15% of the sequences and 2.54% of the base pairs. The DRA accepted 2428 runs of high-throughput sequencing data in 2022. As of September 2023, the DRA provides 16.8 PB of sequencing data in SRA (15.4 PB) and FASTQ (1.4 PB) formats. The GEA accepted 119 submissions of functional genomics data in 2022, totalling 169 experiment datasets via the FTP site (ftp://ftp.ddbj.nig.ac.jp/ddbj_database/gea) as of September 2023. The MetaboBank accepted 14 studies of metabolomics data in 2022, and 109 studies are publicly available via the FTP site (ftp://ftp.ddbj.nig.ac.jp/metabobank) as of September 2023. The JGA accepted 96 studies amounting to 164 TB of data in 2022, and 352 studies, 705 647 samples, 852 TB of human data are available under controlled access as of September 2023. Summaries of JGA studies are available without restriction on the DDBJ Search (https://ddbj.nig.ac.jp/search) and the NBDC (https://humandbs.biosciencedbc.jp/en/data-use/all-researches) website. To access personal raw data, users are required to submit data usage requests to the NBDC. In 2022, there were 194 such requests. An overall statistics is available on our website (https://www.ddbj.nig.ac.jp/statistics/index-e.html).

### MetaboBank

**Figure 1.**
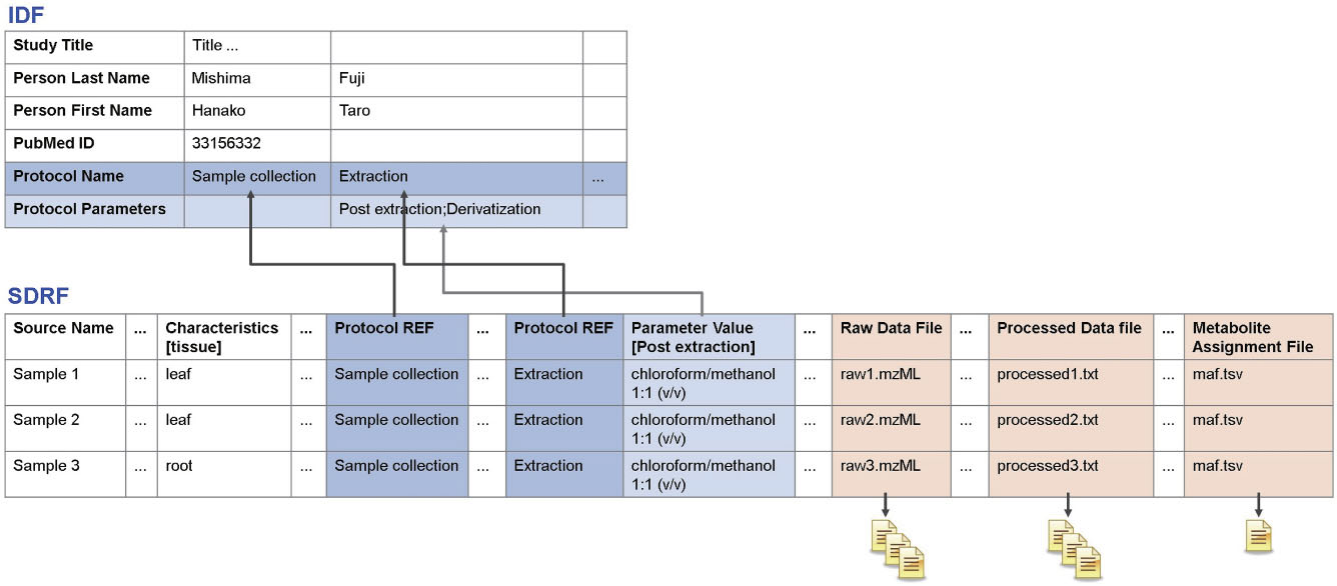
The MetaboBank MAGE-TAB format consists of the IDF and SDRF metadata, raw, processed data files and MAF. IDF provides an overview of the study, protocols, publication and submitter information. SDRF provides sample characteristics and the relation between samples, platforms, raw and processed data files and MAF.

The availability of detailed metadata for experimental measurements is essential for unambiguous interpretation and reproducibility by the wider community of researchers. In addition to experimental raw data and processed data, MetaboBank requires detailed metadata compliant with the recommendations of the Metabolomics Standards Initiative (MSI) (https://metabolomicssociety.org/) (21). To facilitate high-quality but user-friendly submission, the MetaboBank offers Microsoft Excel-based metadata templates covering both mass spectrometry (MS)-based experiments and the nuclear magnetic resonance (NMR)-based experiments. The MS-based templates are further separated into sub-categories: (i) chromatography (e.g. liquid chromatography, gas chromatography), (ii) direct injection (e.g. flow injection analysis, matrix-assisted laser desorption-ionization) and (iii) imaging. Each template consists of two sheets, Investigation Description Format (IDF) and Sample and Data Relationship Format (SDRF) (Figure 1). IDF metadata provides an overview of the experiment, including title, description, experiment type, protocol, publication and submitter details. SDRF metadata provides sample characteristics and the relation between samples, measuring platforms, and raw and processed data files. Completed metadata templates are uploaded together with experimental raw and processed data.

The submission workflow involves five steps: (i) registration of project information to BioProject, (ii) registration of sample information to BioSample, (iii) submission application through the web form, (iv) feedback of a metadata template file filled with information from the registered BioProject and BioSample records and (v) provision of the metadata template, raw and processed data.

Currently, we accept raw experimental data in the form of binary files and/or open-source file formats such as mzML (22) for MS raw data. We also accept a broad range of processed files such as experimental metabolite measurements in the form of concentration, MS peak height or area, retention times and NMR binned areas. We strongly recommend submitters to provide annotated or identified results in the structured Metabolite Assignment File (MAF) format (https://www.ddbj.nig.ac.jp/metabobank/datafile-e.html). The MAF files enable data integration with chemical entities.

**Figure 2.**
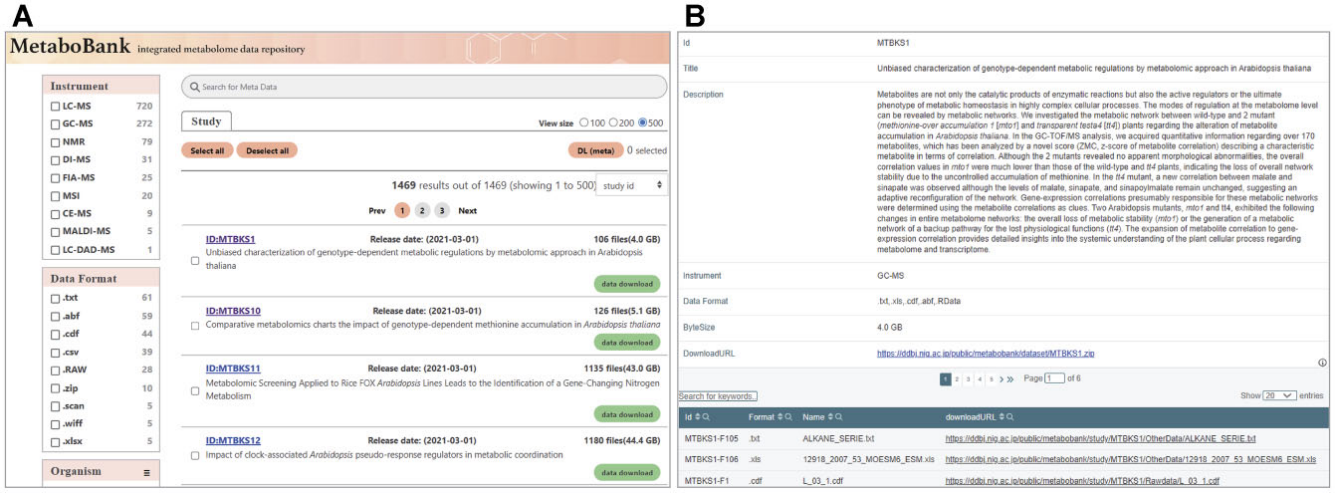
(**A**) The MetaboBank search page. Users can search MetaboBank and MetaboLights studies by free-text and refine the search result using facets. (**B**) The study details page shows the study content and the download links to the metadata and data files.

After the metadata and data files are machine-validated according to the rules (https://www.ddbj.nig.ac.jp/metabobank/validation-e.html), the files are reviewed by curators, and the MetaboBank issues a stable unique accession number with the prefix ‘MTBKS’ to every study (e.g. MTBKS1). The registered data may be kept private for a limited time, typically during the peer-review process of respective publication. A password-protected reviewer access is also available before publication. Once published, metadata and experimental data become accessible at FTP (ftp://ftp.ddbj.nig.ac.jp/metabobank) and the metadata searchable at the MetaboBank search (https://mb2.ddbj.nig.ac.jp/search/, Figure 2).

The MetaboBank search uses a free text throughout underlying data fields, including the study title, description, instrument and data format. The search result page shows study summaries such as the study accession, title, public release date and source repository. Refining the search result is supported by ‘search facets’, to narrow results to a selected instrument, data format and/or organism. Clicking the study accession link shows the study title, description, instrument, data format as well as the download links to the metadata and data files. We are currently collaborating with the MetaboLights (https://www.ebi.ac.uk/metabolights/) repository to implement and explore ways to facilitate metadata exchange in metabolomics. As of September 2023, 104 MetaboBank studies and 1366 MetaboLights studies are indexed.

As metabolome is used in clinical applications, metabolomics data derived from human subjects are submitted to the controlled-access database JGA. As our submission guideline for human data explains (https://www.ddbj.nig.ac.jp/policies-e.html#submission-of-human-data), it is the choice of users to use controlled-access or unrestricted-access databases. To increase visibility and searchability of data, the MetaboBank archives and distributes aggregated metabolomics data (e.g. metabolite concentration among subject groups) as unrestricted-access information. A common BioProject record connects related individual-level JGA data and aggregated MetaboBank data. This integration enables users to search public metabolomics data by keywords such as diseases and biomarkers and to navigate from resulting MetaboBank studies to data usage application of underlying JGA data. The JGA archives metadata and metabolomics data files in the same format as the MetaboBank which allows uniform interpretation and analysis.

## DDBJ system update

### Support of large-scale submission

The MSS (Mass Submission System) application form (https://mss.ddbj.nig.ac.jp/), the registration service for large-scale nucleotide data submissions to DDBJ, is now connected with the prokaryotic genome annotation pipeline DFAST (23). A data submitter only requires a corresponding DFAST job ID for submission instead of uploading the DFAST-annotated results. The form is also connected with the SFTP file upload service to help submitters with large-volume data. The submitter can confirm files and their submission IDs in the history table.

As sequencing technologies are changing and submissions are increasing, our submission processes are also transitioning from manual curation to automatic validation. As part of such efforts, we have automated the DDBJ BioProject submission processes. A submitted BioProject record is validated according to validation rules and the BioProject submission system automatically assigns an accession number to the submitted record if without errors.

### Standard VCF files and the imputation server

In most of JGA whole genome sequencing (WGS) studies, only raw reads in the FASTQ format are registered. We have processed the FASTQ files of selected datasets by using the standard workflows (https://github.com/ddbj/jga-analysis) and provide resulting alignment in BAM files and variant calls in VCF files, so users do not have to perform the two basic analysis steps.

Genotype imputation is a process to infer genotypes of missing variants from specific reference panel datasets. The NBDC-DDBJ imputation server was developed to provide users with a graphical user interface to perform genotype imputation within a secure data analysis environment (24). Reference panels including East Asian-specific panels were constructed by using publicly available 1000 Genomes Project datasets and controlled-access Japanese genotype datasets in JGA for accurate genotype imputation of East Asian populations. The NBDC-DDBJ imputation server is available in the NIG supercomputer and the East Asian-specific reference panels were deposited in JGA and are available once the data usage application is approved by NBDC.

### Supercomputing facility update

The main computing system was installed in March 2019 and consists of a total of 243 computing nodes with 15424 cores; the total computing performance of the CPUs is 434 TFLOPS. In addition, 64 NVIDIA V100 GPUs offer the total performance for double-precision floating-point operations of 499 TFLOPS (https://sc.ddbj.nig.ac.jp/en/guides/hardware/).

The storage system of the NIG supercomputer is divided into two subsystems: one is for database construction and operation at the DDBJ Center (the database storage) and the other is to provide computing resources to researchers (the analysis storage). The analysis part is physically separated into two divisions: general analysis and personal genome analysis divisions.

In April 2023, the database storage was enhanced with a distributed Lustre system of 40 PB which replaced the previous hierarchical system of 12.9PB disk and 15PB tape devices. The database storage will be replaced by the next installation in 2025, when the expected storage size is 60–80 PB. The storage renewal in 2023 is positioned as a preliminary enhancement for gradual expansion of the storage system and data migration. The analysis storage consists of the general analysis and the personal genome analysis divisions, with a total capacity of 17.1 PB.

The supercomputer now supports external workflow execution services (WES) to execute analytical pipelines such as Nextflow, Workflow Description Language, and Common Workflow Language (beta release; https://ddbj.nig.ac.jp/wes/). The DDBJ WES was developed in collaboration with the Database Center for Life Science (DBCLS) on the Sapporo system (25) complying with the Global Alliance for Genomics and Health (GA4GH) WES standard (https://ga4gh.github.io/workflow-execution-service-schemas/docs/). The available pipelines in the DDBJ WES are also published in the DDBJ workflow registry (https://ddbj.github.io/workflow-registry-browser/). It is based on Yevis (26) in compliance with the GA4GH tool registry service (TRS) standard (https://ga4gh.github.io/tool-registry-service-schemas/). The PortablePipeline (https://github.com/c2997108/OpenPortablePipeline) is also available on the NIG supercomputer as a computational engine to perform predefined pipelines on a remote server, including supercomputer system.

## Future direction

To handle the increasing variety of DDBJ services including MetaboBank, we constantly update the common DDBJ account system to accommodate for different submission processes. Integration with other resources such as external databases (e.g. MetaboLights and other INSDC services) is also ongoing. This implies our development of various API services for interoperability.

We also develop the public variation database ‘JVar’ (Japan Variation Database) which is a counterpart of the NCBI dbSNP and dbVar. The JVar will distribute aggregated Japanese variants and frequencies obtained as a result of the JGA raw genome sequencing processing by the standard workflow.

## Data Availability

All resources are publicly available at https://www.ddbj.nig.ac.jp and the data are downloadable at ftp://ftp.ddbj.nig.ac.jp and https://ddbj.nig.ac.jp/public/.
